# Low-Cost Ultrasonic Range Improvements for an Assistive Device

**DOI:** 10.3390/s21124250

**Published:** 2021-06-21

**Authors:** David Abreu, Jonay Toledo, Benito Codina, Arminda Suárez

**Affiliations:** 1 Departamento de Ingeniería Informática y Sistemas, Universidad de La Laguna, 38200 San Cristobal de La Laguna, Spain; jttoledo@ull.es (J.T.); bcodina@ull.es (B.C.); asuper@ull.es (A.S.); 2 Ataman Science S.L.U., 38290 El Rosario, Spain

**Keywords:** visually impaired, ultrasonic, range sensor, electronic aids

## Abstract

To achieve optimal mobility, visually impaired people have to deal with obstacle detection and avoidance challenges. Aside from the broadly adopted white cane, electronic aids have been developed. However, available electronic devices are not extensively used due to their complexity and price. As an effort to improve the existing ones, this work presents the design of a low-cost aid for blind people. A standard low-cost HC-SRF04 ultrasonic range is modified by adding phase modulation in the ultrasonic pulses, allowing it to detect the origin of emission, thus discriminating if the echo pulses come from the same device and avoiding false echoes due to interference from other sources. This improves accuracy and security in areas where different ultrasonic sensors are working simultaneously. The final device, based on users and trainers feedback for the design, works with the user’s own mobile phone, easing utilization and lowering manufacturing costs. The device was tested with a set of twenty blind persons carrying out a travel experiment and satisfaction survey. The main results showed a change in total involuntary contacts with unknown obstacles and high user satisfaction. Hence, we conclude that the device can fill a gap in the mobility aids and reduce feelings of insecurity amongst the blind.

## 1. Introduction

The number of totally blind people along the world continues growing over the years. There were 39 million in 2010 according to World Health Organization [1]. Including persons with low vision (visual acuity from 6/18 to 6/60 [2]), the number rises to over 285 million. One of the implications about visual loss is the limitation in independent mobility [3]. The three elements that determine autonomous travel of the visually impaired are: (i) safety (involuntary contacts), (ii) efficiency (spatial orientation) maintenance, and (iii) fluency (walking speed) [4]. Thus, blind persons can move autonomously in structured and known environments. However, new places are usually challenging. Even in known environments, safety is compromised by obstacles that have changed location and moving elements such us pedestrians.

Among blind people, there are two popular travel aids: white canes and guide dogs [5]. Each person has to learn and adapt their habits to the chosen aid, and their autonomy depends on the skills they can develop. The white cane is quite well known, cheap, and provides really valuable information about the environment. However, cane length limits user ability. Detection projections and hanging obstacles are very difficult and users often have collisions. On the other hand, a guide dog is expensive and requires high care. Most blind people can not afford one, and the waiting time to get one is quite long.

The bibliography shows many attempts to solve this problem with engineering approaches, but despite the several prototypes and years of research with Electronic Travel Aids (ETA) [6] as a solution to improve capabilities of blind people using different sensors [7,8], none of the proposed solutions have shown widespread adoption. Some of the reasons are the high price and the fact that they are difficult to use.

This project was conducted from user perspective to avoid falling into the same problems as previous researches, focusing on user satisfaction and keeping the device low-budget, wearable, simple and robust against failures and interference [9].

The device to be designed must also improve safety of the white cane, detecting not only obstacles in the ground but also at head level. Thus, instead of a cane replacement, the design should be used as a complementary tool. However, it should also be possible to be used standalone, for example, by low-vision people who do not use a white cane as a safety device. In this case, a small and unnoticed device should be designed.

Requirements for the device should then include: (i) low budget as the principal aim, (ii) detection of low and high obstacles, (iii) robust against failures and other devices and (iv) easy to use. Some research related with the user experience and functionality has already been done with the eBAT [10].

In this paper, the sensor design is presented, choosing parts and devices so as to obtain a low-cost sensor that is reliable and easy to use. The paper is structured as follows: in Section 2, similar devices presented in the bibliography are studied. Section 3 studies the selection components, elements, and programming to obtain a low-cost and robust prototype. Section 4.4 presents a set of results with actual blind people, and in Section 5 some conclusions are drawn.

## 2. Related Work

Several ETAs have been developed by different authors.

One of the commercially available ETAs is the Miniguide [11]. With a torch form factor, it is placed in the hand and pointed towards the walking path. Measuring distances with an ultrasonic sensor, it provides haptic or sound feedback. It is priced at USD 499 (https://lssproducts.com/miniguide-mobility-aid/ accessed on 20 June 2021).

Ultracane is also another commercial product, based on a regular white cane. With two ultrasonic sensors, it can detect objects both in the walking path and head/chest height. Feedback comes from two vibrating buttons in the handle. The price is GBP 590 (https://www.ultracane.com/ultracanecat/ultracane accessed on 20 June 2021).

The Buzzclip is a simple device that uses one ultrasonic sensor to measure distance to obstacles and gives back vibrations on the device. It has a clip to place it in different locations. The price is USD 249 (https://www.imerciv.com/index.shtml accessed on 20 June 2021).

Similar to the Buzzclip, the iSonar, developed by Vorapatratorn & Nambunmee [12], uses one ultrasonic range sensor to warn visually impaired people about obstacles in the surroundings. It is worn around the neck and gives two options for the feedback: warning sound and vibration. The vibrating motor is placed in the device, so the haptic feedback is perceived with the contact to the skin.

Gayathri et al. [13] developed an improved version of the white cane, including sensors to detect bumps in the floor and the presence of water. Feedback is provided using sounds.

Sonarglasses (https://sonarglasses.com/sonar-glasses/ accessed on 20 June 2021) is another commercially available device that uses ultrasounds to give vibrations. It has a form factor of regular glasses. Price is USD 799.

Katzschmann et al. [14] presented a belt covered by seven infrared time-of-flight sensors. When an obstacle is detected, the user receives feedback in the form of vibrations. The haptic signal is provided by a set of motors in a strap placed on the upper abdomen. Each sensor have a cost of 125 EUR; thus the minimum estimation for the device is about 900 EUR.

Ramadhan [15] recently developed an ETA that includes an ultrasonic sensor to detect obstacles and can provide three kinds of alarms. Sound or vibrations are the options for the feedback to the user. It also provides GPS tracking to a phone using on-board GSM connection. It is intended to alert relatives when the visually impaired is in any difficult situation.

All the devices present in the market have some problems, including size, weight, battery duration, reliability, being difficult to use, price and being hard to learn [16]. The authors note that none of the previous designed devices has been broadly adopted, and there is no one that can be labeled as reference aid. Thus, there is still room for new products.

One of the main problems in the market for devices is the price. In this paper, a very low-cost ultrasonic range ETA is presented. The prototype is designed based on a low-budget schema but including some characteristics that none of the previous devices includes, such as the capacity for detecting if the received ultrasound echo comes from the device itself, improving the robustness of the sensor. A phase modulation is included in the ultrasonic wave in order to detect the origin of the data echo without increasing the price. This ultrasonic phase modulation has been also studied and tested [17,18], however, using complex and expensive devices that can not be included in a low-cost ETA category.

## 3. Materials and Methods

### 3.1. Range Sensor

There are several types of range sensors that can be divided into three families according to measurement technology: optical, radio-frequency (RF) and ultrasonic, all with advantages and disadvantages.

In the optical family, laser and infrared are the main ones. The highest accuracy is found with the laser units, but they are usually expensive and the measurement is punctual, with a very small detection angle [19]. Those based on infrared diffuse light do not have enough power to properly work outdoors. RF sensors are still under development for short ranges but are starting to be competitive.

Keeping in mind that low budget is a priority of this design, the selected sensor family was the ultrasonic, based on prices and the reasonable accuracy that can be achieved. They can work outdoors and indoors with a quite large detection angle to perceive obstacles from different directions.

**Figure 1:**
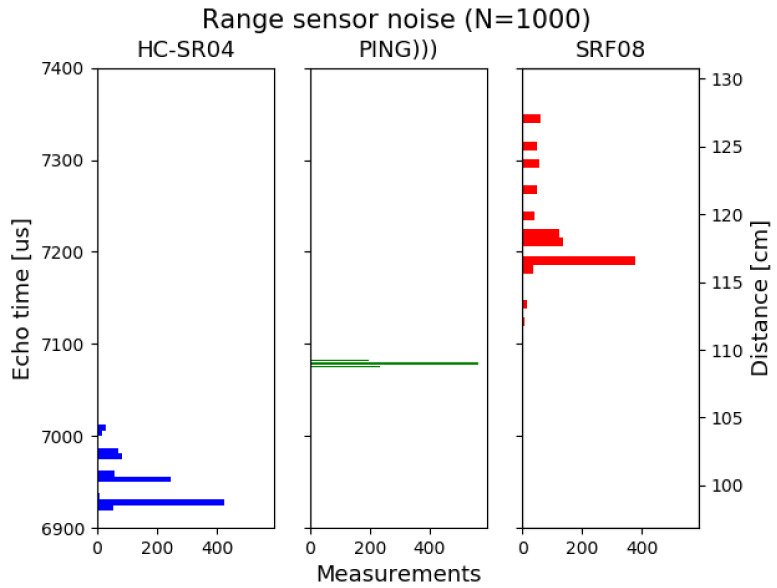
Measurement noise to an static obstacle with a HC-SR04 sensor, a Parallax Ping sensor and a SRF08 sensor.

Several models of ultrasonic distance sensors were tested. In Figure 1, the result of 1000 measurement to a 1 m obstacle is shown for an HC-SR04, a Parallax Ping and an SRF08 ultrasonic sensor. Results show that Parallalx Ping is the best one, with a normal distribution centered on 1 m but with a unit price of about USD 30. HC-SR04 has reasonable accuracy, but the measurements tend to underestimate the actual distance, and the dispersion is larger. However, it is the sensor with the lowest price (USD 2). The worst results come from SRF08, with greater measurement dispersion than others and a price of USD 25. For this project, and keeping low budget in mind, HC-SR04 has demonstrated enough accuracy and a great value–price relationship. Two of them are needed to detect obstacles from both the ground and the head level, so the unit price is critical. The selected model is then the low-cost HC-SR04 [20]. The main characteristics of this device are its low budget, being widely tested and used, low power consumption, and fifteen-degree measuring angle. It provides a detection range from a few centimeters up to four meters.

The HC-SR04 emits 8 × 40 kHz pulses, falling in the ultrasound range beyond human hearing [21] but within the limit for dogs [22], which can go as far as 47 kHz. Some tests were done with the range sensor and guide dogs and no disturbance was observed. Despite this, further research should be done to verify that the ultrasonic waves do not annoy them.

**Figure 2:**
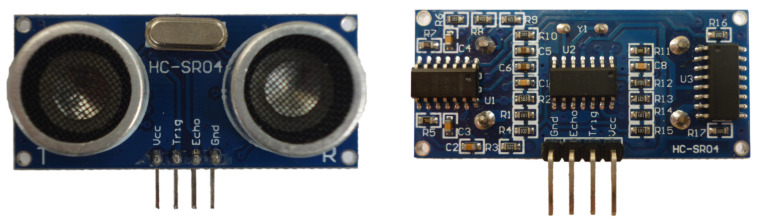
Picture of the HC-SR04 ultrasonic sensor. Top view in the left panel, bottom view in the right panel. The ultrasound emitter is labeled as “T” (**left**) and the receiver as “R” (**right**). It has 4 pins: “Vcc” and “Gnd” to provide the needed power, “Trig” to deliver the trigger signal that activates the ultrasound emission, and Echo”, which is activated when the echo is detected.

A picture of the HC-SR04 range sensor can be found in Figure 2.

The HC-SR04 includes two transducers: one for ultrasonic emission and the other for detection, with a band centered at 40 kHz. It provides four pins for external device control: two of them to power the unit, one to trigger the ultrasonic signal and the last one that rises until the echo is detected. The HC-SR04 receives the measurement commands with a pulse in the trigger signal, and then it sends the ultrasonic pulses and listen for the echos. All the electronics to generate the ultrasound pulses, the signal amplifier and a level comparator are included in the board. The HC-SR04 is used as a base design platform, with its characteristics modified and its software performance improved with minimum changes in the hardware.

The method used to calculate the distance to the objects detected is the Time-of-Flight (ToF) [23]. By measuring the time it takes between the sound waves’ emission and the echo detection, and knowing speed of the sound in the environment (*c*), distance (*D*) can be obtained according to the Equation (1).

(1) $$D=\frac{ToF}{2}c$$

where ToF is the time difference between emission of the sound wave and the reception of the echo. However, the speed of sound is not constant.

#### 3.1.1. Speed of Sound

**Figure 3:**
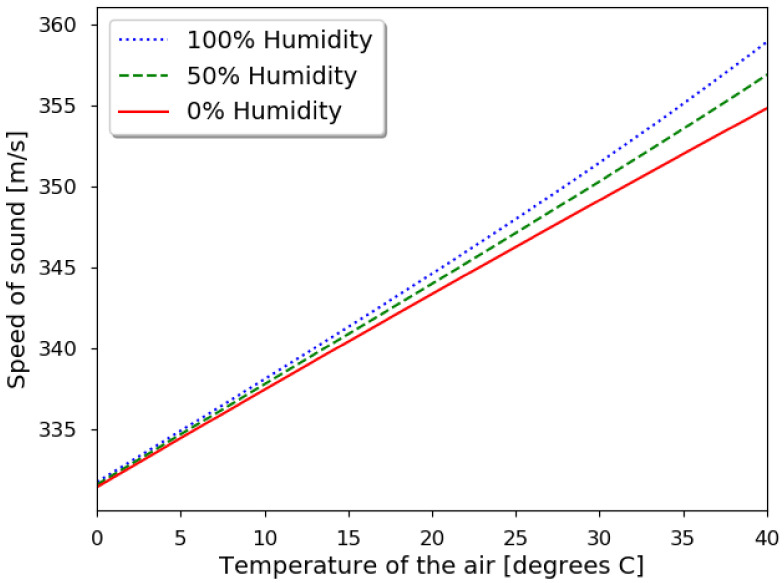
Speed of the sound in the air for different temperatures and three humidity levels.

In general, the speed of sound in the air is described by the Boyle’s law [24], expressed by Equation (2).

(2) $$c=\sqrt{\frac{\gamma_{s}RT}{M}}$$

where $\gamma_{s}$ is the specific heat ratio, *R* the universal gas constant, *T* the absolute temperature (in Kelvin), and *M* is the molar mass. From the formulation, it can be derived that there is dependency on the temperature and the humidity [24,25]. Graphical representation of this variability is shown in Figure 3.

Around room temperature (20 °C), the variation with air temperature is almost linear, and the influence of the humidity is quite contained. In the case that this speed is used to measure a distance, placing an obstacle at one meter can give different results. A variation of 10 °C gives a 3.5 cm error. To compare, for humidity, the difference between 0% and 100% is 0.7 cm. Thus, assuming room temperature and 0% humidity can give an error of 4.2%.

These source of errors come from the natural propagation of sound waves and can not be avoided but can be corrected using environment sensors. For the approach of this study, the error is assumed because distances lower than 10 cm are negligible from the human point of view.

#### 3.1.2. Sensor Signals

**Figure 4:**
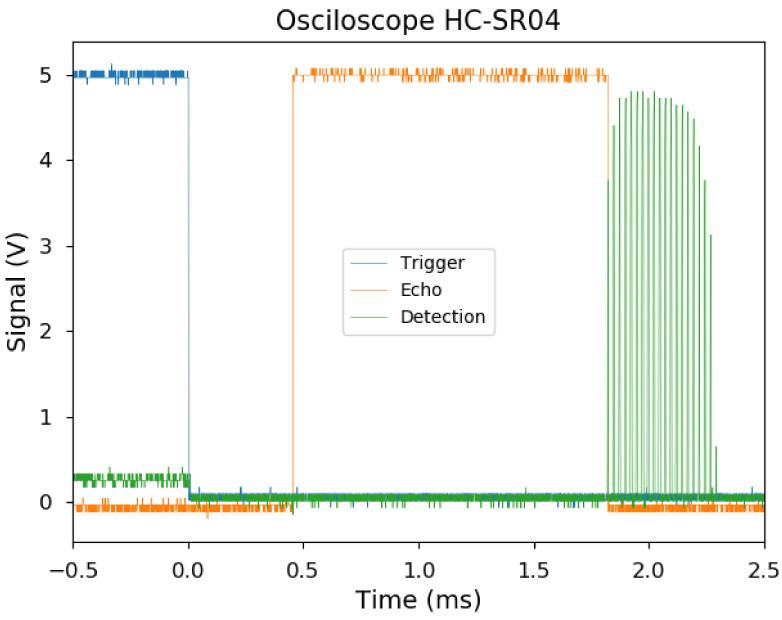
Signal obtained from HC-SR04 “Trig” pin (blue line), “Echo” pin (orange) and detection (green) of a flat object at 30 cm.

To better understand the behavior of the HC-SR04 sensor, an oscilloscope was connected to pins labeled “Trig” and “Echo”, and the detected signal was obtained from the comparator circuit. Signals obtained for a flat object at 30 cm are shown in Figure 4.

The “Echo” signal rises when the HC-SR04 changes its mode from emission to detection until any signal is received back.

Figure 4 shows that there is an offset of 0.453 ms between the trigger (blue line) and the echo (orange line). This corresponds to the 8 × 40 kHz pulses (0.4 ms) emitted, avoiding confusing the receiver. For room conditions it means 7 cm.

In the detected signal (green line), the individual pulses of 0.025 ms (corresponding to 40 kHz emission) can be observed. First peaks are weaker as the piezo is starting to vibrate. Then, after the 8 pulses, the piezo keeps vibrating and generating additional weaker peaks. Orange line represents the detection time, starts after emitting the pulses and ends with the first one received.

#### 3.1.3. Sensor Stability

**Figure 5:**
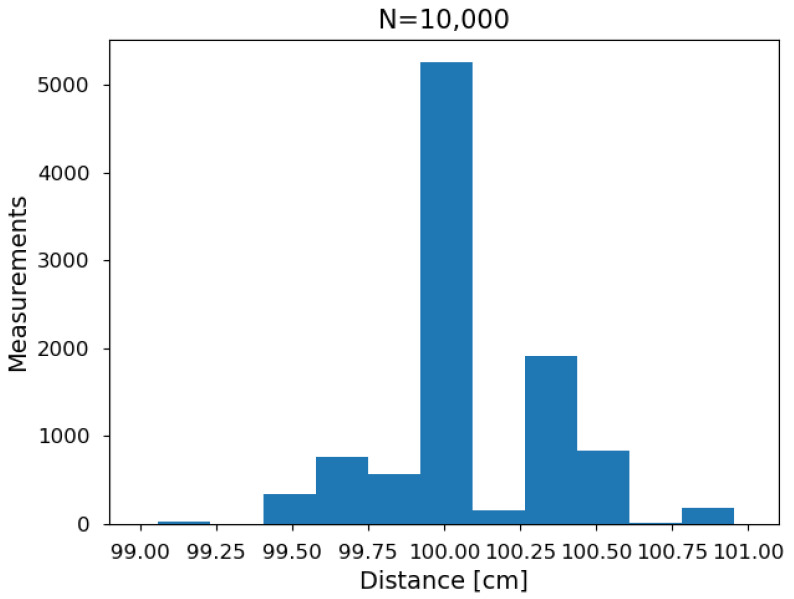
Histogram of 10,000 distance measurements of static obstacle located at one meter from the HC-SR04 sensor. It is centered at 100 cm and spreads over a bit less than two centimeters with 0.269 standard deviation.

Another error source when measuring distances with a range sensor is the stability. To characterize the noise in the HC-SR04, a flat panel was placed in front of the sensor at a fixed distance of one meter. One measure each second was acquired to a total of ten thousand. The histogram of the recorded values is presented in Figure 5.

The results show a distribution centered in 100 cm with an standard deviation of 0.269. Values move from minimum to maximum over less than two centimeters.

Applying a normal test, a value of 114 is obtained with a *p*-value $1.46\times 10^{-25}$, allowing Gaussian behavior to be rejected. A test for unimodality [26] gives a result of D=0.09035 with *p*-value < $2.2\times 10^{-16}$. This rejects the unimodal assumption, meaning that the distribution is at least bi-modal.

Looking at the signal obtained from the sensor (Section 3.1.2), the peaks can be explained by different pulses detected. The 40 kHz emission (0.025 ms) correspond to 0.43 cm. Then, an obstacle at one meter is few times detected by first (low energy) pulses. Most of the times by the stronger signal (central peak). And then some more times by the following vibrations of the piezo, corresponding to peaks at 100.4 and 100.9 cm.

Compared with better-quality ultrasonic range sensors [27], the noise is a bit higher. However, value for money is on the side of the HC-SR04 as other sensors are more than 20 times the price. In any case, the recorded noise is sufficiently small for obstacle detection as it remains below 1%.

#### 3.1.4. Measuring Angle

One characteristic related also to ultrasonic sensors is the detection angle. Although the manufacturer provides a value of 15 degrees, this number should be different for diverse obstacles and can even depend on their shape.

**Figure 6:**
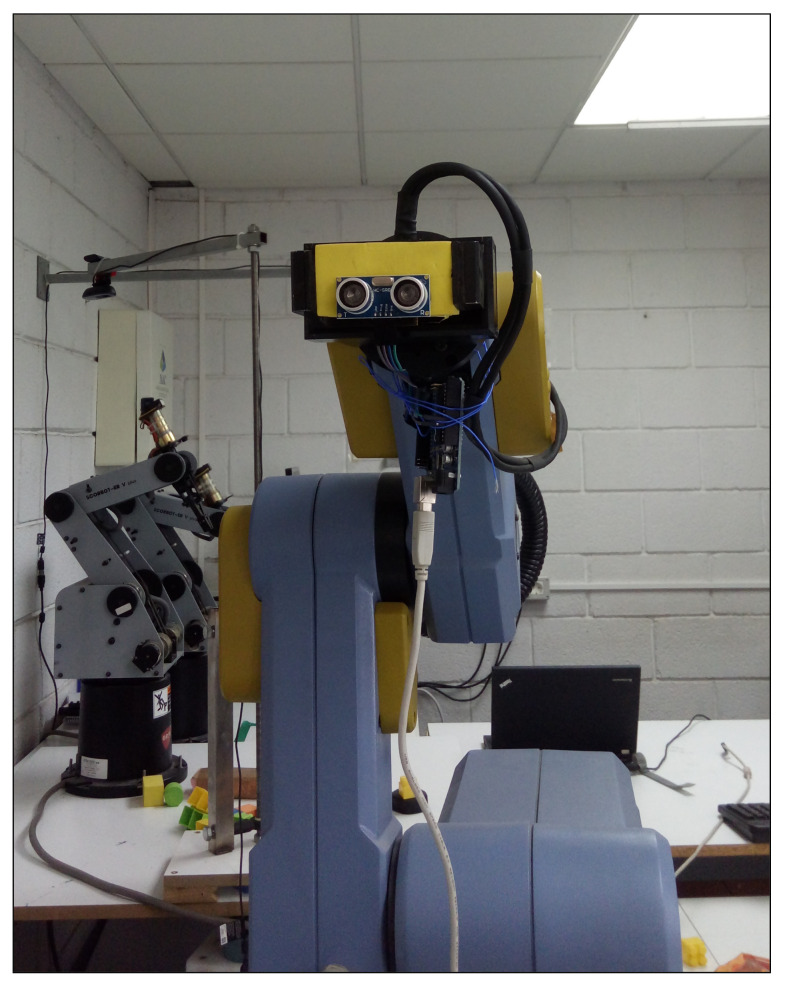
Picture of the laboratory setup to test the aperture of the HC-SR04. Range sensor is mounted in the robotic arm that can rotate the base with high accuracy, leaving the other axis fixed. Data are acquired using an Arduino UNO board (under the sensor) and serial monitor.

To validate sensor’s behavior, a configuration was set up in the laboratory using a robotic arm. See picture in Figure 6.

**Figure 7:**
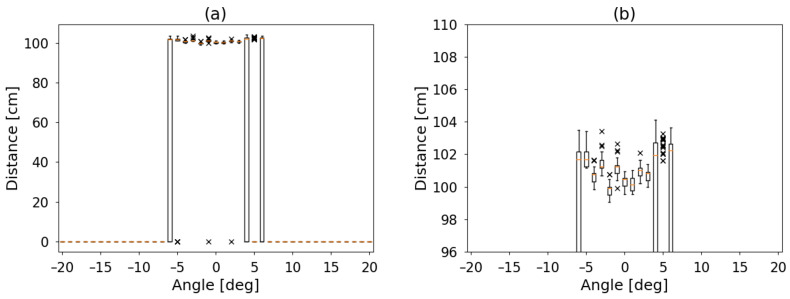
Measurement results for a cylinder one meter height, 75 mm diameter located one meter from the sensor. For steps of 1 degree, 100 measurements were acquired each. (**a**) In the left panel, the box plot of the data is in full range. (**b**) In hte right panel, details of the values where a detection was performed. The bottom line of each box indicates the first quartile. The box itself is the second and third divided by an horizontal line (the median). The top line is the fourth quartile. Outliers are plotted as individual points with a cross. The obstacle is detected from −6 to +6 degrees with a dispersion of 4 cm.

**Figure 8:**
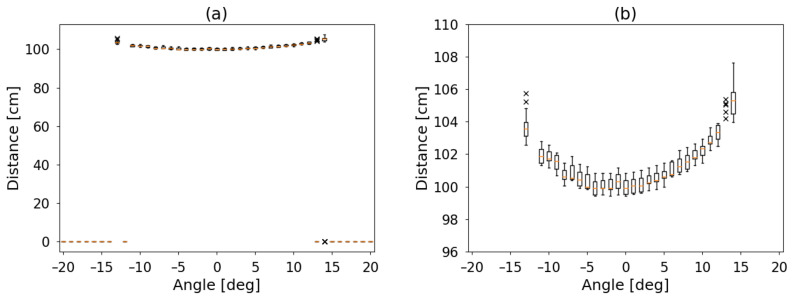
Similar to Figure 7 for a cylinder one meter height and 120 mm of diameter. (**a**) Full range in the left panel. (**b**) values where a detection was performed in the right one. The dispersion for this larger obstacle is lower, and the round shape of the cylinder can be observed. It can also be observed that the detection is not horizontally symmetrical, as the range sensor has the ultrasound emitter on one side and the detector next to it. The object was detected for angles between −13 and +14 degrees and 2 cm dispersion.

For steps of one degree, a set of one hundred measurements was acquired. The process was done for two plastic cylinders located at one meter, both one meter in height but with two diameters: 75 and 120 mm. Measured distances are displayed in Figure 7 and Figure 8, using value of 0 cm when no detection was performed.

For both cylinders, a box plot [28] is shown. There, each set is described by a box with a central horizontal line and two vertical lines. From the bottom to the box, the first quartile. The box is divided by the median into the second and the third quartile and the top line up to the fourth quartile. Outliers are identified as individual points with a cross symbol.

**Figure 9:**
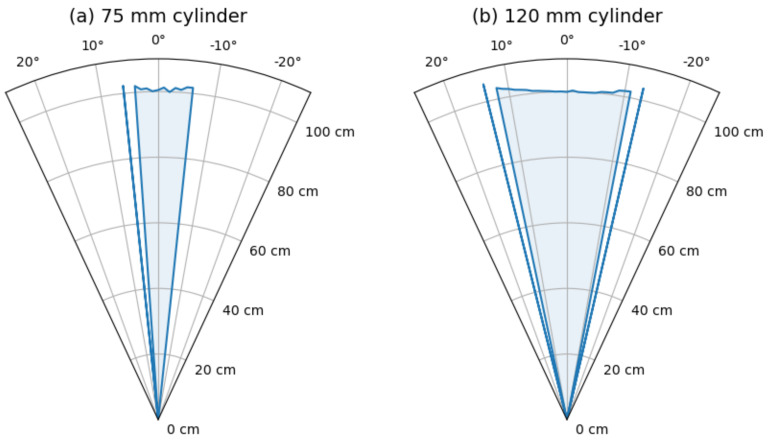
Polar plot of median values for each angle. (**a**) Left panel for the 75 mm cylinder. (**b**) Right panel for the 120 mm.

Left panel of Figure 7 and Figure 8 show the full range of data and right panel the detail where something was detected (non-zero). For better comparison, a polar plot of median values is shown in Figure 9.

Can be observed that the detection angle for the small object is about 12 degrees and 25 for the larger one. The detection is also not symmetric. This last is not unusual, as the detector itself has two transducers with different tasks next to each other. However, the profile of the cylinder can be intuited.

**Figure 10:**
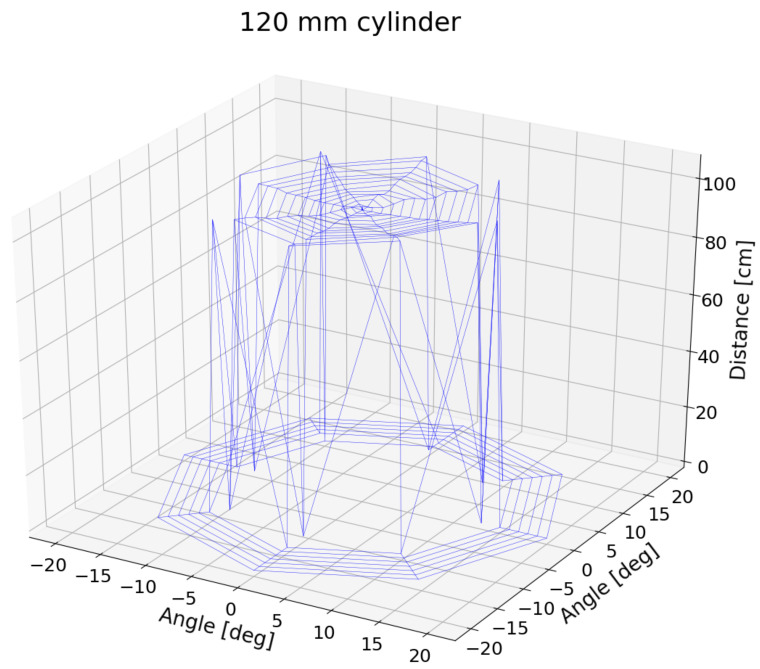
HC-SR04 detection of the 120 mm cylinder. For wrist angles of −45, 0, 45, and 90 degrees of the robotic arm, an scan of steps of one degree around z-axis was performed.

To continue with the sensor inspection, for the large cylinder, data were acquired for three more orientations. As already done for the HC-SR04 aligned with the horizon (0 degrees), −45, 45, and 90 degrees were added, which were obtained by rotating the wrist of the robotic arm and scanning the obstacle again for steps of one degree around z-axis. The 3D plot of different orientations is presented in Figure 10.

Despite some differences on the edges of the detection, the conical shape of the sensor emission-detection can be assumed. There is no preferred orientation to improve the signal.

This sensor in particular is quite directional compared with those studied in other works [29,30]. Although the sensibility is not as good as in other high quality sensors [30], it is sufficient to fulfill the requirements of this study.

The lower detection angle is an advantage in this case, providing a better compromise between detecting obstacles in different directions and those outside the travel path, because the reflections coming from the last ones are reduced. The sensibility is again justified by the reduced cost of the HC-SR04 units.

### 3.2. HC-SR04 Working Principles

**Figure 11:**
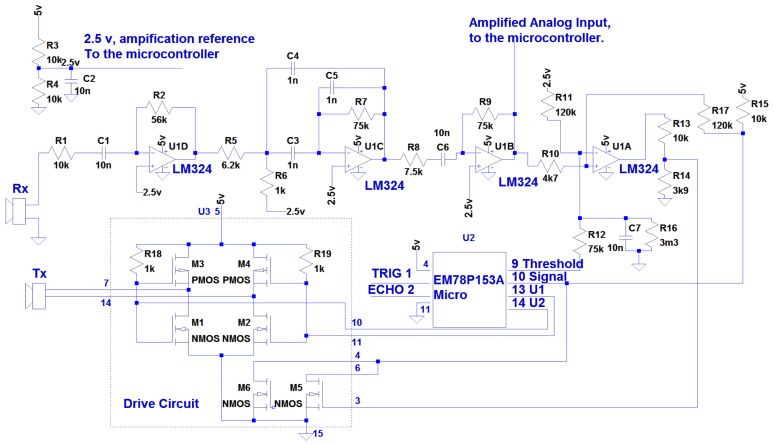
HC-SR04 circuit Diagram.

In Figure 11, a general schematic of the HC-SR04 is shown. The ultrasonic range is composed of three circuits, a LM324 classical operational amplifier (circuit U1), a specific unmarked drive circuit as power amplifier and a signal adapter (circuit U3) and a EM78P153 8 bit One-Time Programming (OTP) micro-controller (circuit U2).

The module is divided in two parts, the ultrasound reception, amplification and detection and the ultrasound generation. All the steps are controller by the EM78P153 micro-controller.

In the ultrasound reception, the transducer RX is a piezo electric sensor centered on a 40 kHz bandwidth. It listens to the ultrasounds in the environment and generates a millivolts range signal (from 5 to 50 mv). This input signal is amplified in the first step by the U1D operational amplifier, configured as inverting amplifier, with a gain of 5.6. The output voltage is centered at 2.5 volts, generated by a voltage divider based on the 5 v voltage input (R3 and R4 resistor). The next step is a band-pass filter in the U1C operational amplifier; the band pass filter is centered at 12 kHz, which looks like as a design error. This filter should remove noise filtering for frequencies other than 40 kHz, but the error in the design converts this stage into a signal loss. The last step is an inverting operational amplified centered at 2.5 volts, with a gain of 10. After these steps, a filtered amplified signal, centered at 2.5 volts is obtained. This signal has a peak-to-peak voltage from from 0.1 to 0.5 volts, depending on the distance and the material of the detected obstacle.

The next step in the HC-SR04 is detection. Detection is made based on the operational amplifier U1A in a comparator configuration. The sensitivity of the detector can be changed using the threshold pin [31]. If the threshold input (pin 9 of the EM78P153 micro-controller) is fixed to high impedance, this means that the comparator is working on maximum sensitivity. When the threshold signal is set to 0, the value of comparisons is reduced to apprimately 1 volt, and the sensitivity of the comparator is reduced to the minimum (a signal of 1.5 volts peak to peak is needed to activate the comparator). A pulse of 500 µs is applied in the threshold signal by the micro controller to avoid the detection of the emitted signal as an echo; in that time, the C7 capacitor discharges, generating a minimum value of 1.3 volts (which means that a received signal centered on 2.5 volts should achieve 1.3 volts to activate the comparator, 1.2 volts of peak to peak signal). When the threshold value is fixed to high impedance again, the comparison value increases to a value of 2.3 volts in 3 ms (a signal of 0.2 volts peak to peak is enough to trigger the comparator). This generates a comparison ramp, avoiding the detection of low-power close signals and increasing sensitivity over time. Any signal smaller than 2.3 volts in the static, or the ramp voltage when capacitor is charging (between 1.3 and 2.3 volts), will trigger the comparator, and a detection will be found. The output of the U1A operational amplifier is converted to a 5 volts TTL signal using the internal transistor inside the Drive Circuit (circuit U3). The operational amplifier gives 0 when echo is detected; this signal is applied to the base of the transistors inside the U3 circuit. This allows it to convert the analog output signal to a 5v TTL signal received by the EM78P153 micro-controller and used to stop the detection counter.

The generation of the ultrasonic wave is straightforward. The EM78P153 micro-controller generates a train of eight pulses using two pins in an inverting way. When pin 13 is 5v, pin 14 will be 0 and vice versa. This signal is used to polarize the H-bridge inside U3, getting a 10 volts 40 kHz signal, applied to the TX piezo electric transmitter.

The sequence of steps on a conventional HC-SR04 sensor is described in Figure 4 and is based on the following steps:

1. The master microprocessor, which controls the HC-SR04, generates a pulse in the Trigger pin of the connector (pin 4 of the EM78P153 micro-controller). This starts the measurement process.
2. The threshold pin is set to 0 to avoid detecting the pulses emitted by the TX.
3. The EM78P153 generates 8 pulses of 40 kHz, in its 13th and 14th pins (U1, U2). These pulses are amplified in current and voltage by the H bridge of the Drive Circuit, and applied to the TX ultrasonic emitter.
4. The Threshold pulse is fixed to high impedance, 10 µs after last pulse of 40 kHz is emitted. This allows the C7 capacitor to charge and generates a dynamic threshold for the UA1 comparator. It takes about 3 ms to get the value of 2.3 volts for comparison. This prevents false positives due to detecting emission waves as reception.
5. The Echo signal is set to high, and the time measurement to obtain the distance begins.
6. When a signal smaller than threshold value is received, the comparator is triggered, and activates the M5, and M6 transistors inside the Drive Circuit. This convert this signal into a TTL signal, received by the EM78P153. When the first detection pulse is received, the Echo signal is set to 0, indicating that the measurement is finished. The time that Echo pulse is high, is proportional to the obstacle distance and the microprocessor which controls the HC-SR04 can use this time to measure the ultrasonic travel time.

### 3.3. HC-SR04 Modifications

HC-SR04 is quite cheap, and an external microcontroller is needed to use it. In the eBAT design, the standard ultrasonic receiver is modified to improve accuracy while maintaining the low cost. It is the cheapest option available, and the results can outperform more expensive options.

The first step consists in changing the badly tuned band pass filter, centered at 12 kHz. Changing R5 to 2.5 KΩ and R7 to 22 KΩ, the band pass filter will be centered at 40 kHz. This change allows it to obtain an output signal on U1B 10 times bigger than before, allowing it to detect not only distant obstacles but small close obstacles.

One of the problems of the eBAT system is the interference between two prototypes. This device is designed to help blind people, and users will meet frequently. Blind people share associations and areas, and the possibility of interference between different eBAT devices is large. Other sources of interference can include car parking ultrasound sensors and similar devices. A conventional ultrasound measurement system sends an ultrasound train pulse and wait for a response. The system does not discriminate if the response comes from other source, and that can produce errors. To avoid this problem, the device has been modified to detect echos coming only from its own source.

The idea is to identify the received ultrasound pulses. For that purpose, the emitted pulses should be representative. One way to identify the pulses is modulating information in them. The modulation can include a code that can be used to distinguish the emission source. If the received pulses are not detected as the ones emitted, because code is erroneous or modulation is not detected, the echo will be discarded. If this happens 3 consecutive times, the user will be warned, that the results can be inaccurate due to the presence of other ultrasound source.

To include information in the ultrasonic wave, a modulation should be applied. There are three main kinds of modulations: amplitude, frequency, and phase. Amplitude modulation cannot be used, as the received signal amplitude can be affected by many parameters such as obstacle size, distance or surface. The ultrasound emitter and receiver maximum performance is fixed to 40 kHz, so a change in frequency reduces accuracy and range, and it implies a change in amplitude. Thus, the natural way to include codification in a ultrasound pulse train is to code information, changing the phase in a Phase-Shift Keying (PSK) style modulation. To distinguish this phase change, a 180-degree change is used in order to maximize code detection. In [18], a similar schema is explained but with a very complicated and expensive hardware that is quite difficult to implement in an actual application.

The PSK modulation used is DPSK. DPSK is a digital modulation based on changing phase of a sinusoidal signal. In this case, the 40 kHz ultrasound frequency carrier is used. The phase of the signal changes 180 degrees with every bit transmitted, so a change in the phase means a change in the transmitter bits, and no change means the bits keep constant. The received will obtain the phase changes in the carrier, and it detects the transmitter bits. DPSK is simpler to implement than ordinary PSK, as it is a “non-coherent” demodulator. There is no need for the demodulator to keep track of a reference wave, and demodulation is made that only compares phase changes.

To make these changes possible, the EM78P153 OTP micro controller is removed from the board, and the 2.5 volts reference, U1B operational amplifier output, and U1 and U2 ultrasonic generation are directly connected to an ATMega328P, low-cost micro controller in an Arduino Nano board. All the measurement tasks, ultrasound wave generation, and blind user interface are made in the same micro controller, thereby reducing the costs. The tasks made in the micro controller are:

1. 40 kHz ultrasound wave generation with PSK code modulation.
2. Reception of ultrasound echos and measurement echo time.
3. Signal reception demodulation and source verification.
4. User interface.

**Figure 12:**
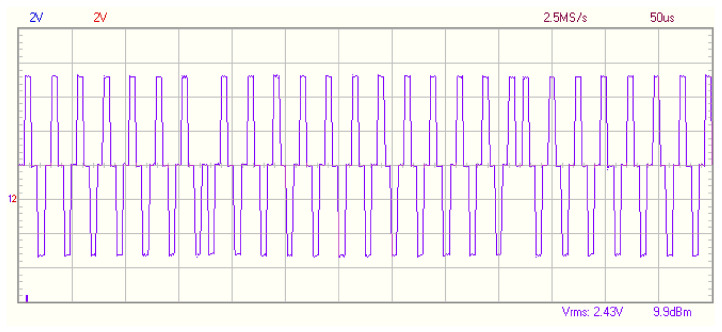
Modified sine wave modulation.

For step 1, the micro controller generates a 40 kHz signal codified with DPSK information. It is easy to modulate analog signal as in [18]; however, a high-speed digital analog converter is expensive. Therefore, an analog modulation should be made using only digital available signals. In this case, a modified sine wave of 40 kHz is used, as shown in Figure 12. This digital signal simulates the behavior of a true sine wave. The piezo electric emitter converts this digitized signal into a analog one due to its dynamics. Only two digital pins are used to generate it, using a very accurate time control. In the sequence presented on Figure 12, three different phases are presented, it starts with a normal phase, and after 7 pulses, a 180-degree phase change is generated, and again after 11 pulses, the original phase is emitted. With this train of pulses, applied to an analog piezo electric emitter, an actual analog sinusoidal wave with different phase modulation is emitted.

**Figure 13:**
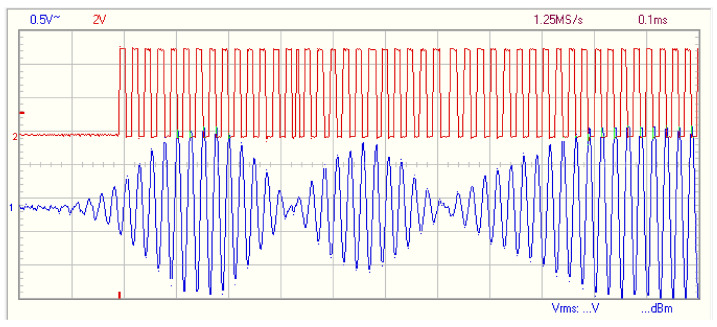
Received signal after obstacle detection.

For step 2, the receiver transducer listens to the environment, getting all the ultrasonic information. The aspect of a received modulated signal is shown in Figure 13 as a blue plot. The time of the first pulse when the received wave is bigger than a dynamic threshold is saved. This threshold decreases over time, controlled by a micro controller software. After this time, the output of the internal analog comparator is shown in red. A temporal filter is applied after the first pulse in order to avoid possible noise, and a inverse (negative or positive) pulse of similar amplitude should be received after approx 12.5 µs. The threshold is calculated based on the average analog input value before sending the transmission pulses. This whole process is completed in the micro controller program, and no external components are needed. The in-circuit analog-to-digital converter is used to measure the signal level and is set to a 6.5 µs measurement time, reducing the conversion resolution to 8 bit in order to increase measurement speed. Using a frequency measurement of 150 kHz, the 40 kHz input signal is correctly sampled according to the Nyquist–Shannon theorem. In this step, a signal is received and a distance can be calculated, but now the modulation is tested to detect the signal source. The red plot shown in Figure 13 is saved using the times when the received signal changes from 0 to 1. The time in µs when the signal change is stored, allowing the entire reception to be saved. This saves computation time, as signal reception and processing can be made at different times.

For step 3, the received signal demodulation is made in order to detect the origin of the ultrasound pulses. The saved signal in step 2 is processed in order to detect if it comes from the emitter, so a valid detection is achieved. In the example of Figure 13, there are three phase changes in the signal. To detect the signal in a low-cost micro controller, the high speed in the circuit analog comparator is used. The comparison is made with respect to the middle voltage point of the amplification coming from the ultrasound board, as shown in Figure 11. The binary generated function is shown in red in Figure 13, and it can be seen that the phase change is also appreciable in this digital signal. In the micro controller, a digital phase detection is made. This detection is quite easy, and it does not consume many computation resources. The demodulation is made based on a XOR comparison between 0-degree phase signal, and the received signal. This comparison obtains the phase change of the received signal over time.

The comparator included in the HC-SR04 circuit is no longer used, and it is not necessary to detect the obstacles. The continuous analysis of the received signal filters any glitches or noises that could trigger the comparator, obtaining a more robust and accurate detection.

**Figure 14:**
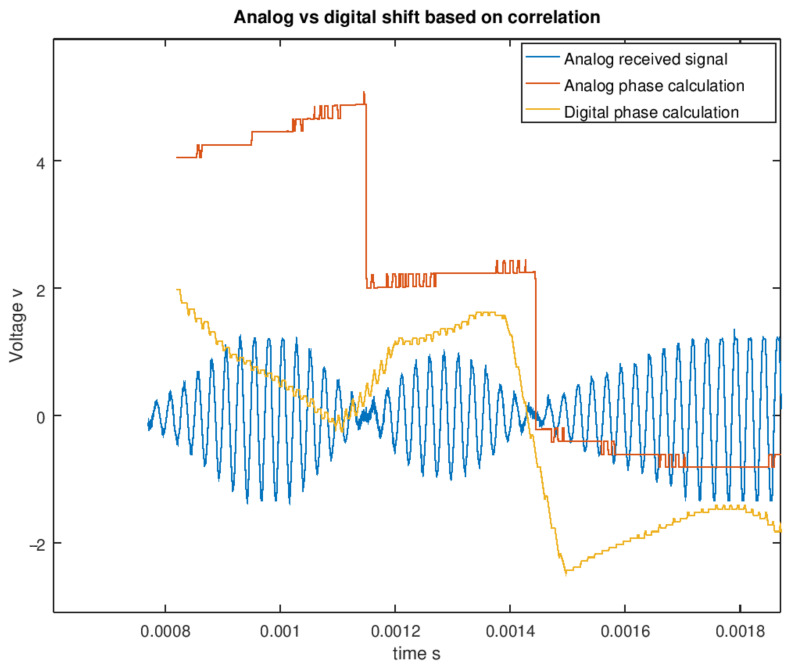
Signal demodulation and code extraction.

In Figure 14, the demodulation of the received signal is presented, where blue plot shows the captured analog signal, (blue plot in Figure 13), the yellow plot shows the demodulation of the analog signal using the phase change detection, and red plot shows the demodulation of the digital signal (red plot in Figure 13). The code included in the emission phase is recover in the demodulation of the binary signal. The maximum bit code rate is about 3.000 bps, but in this case, a short code is sent to avoid reducing minimum obstacle detection distance. As we can see, it is quite simple to detect the code in the analog and digital demodulated signal. If the received signal does not match with the emitted code, it will be detected as an error, and the distance will not be valid. If the code of the demodulated signal does not match, it will be detected as an interference or a measurement error.

As a downside, while the demodulated signal is emitted (1.7 ms), no detection can be performed. This means that objects closer than 28 cm can not be detected by the sensor. Fortunately, this distance is under the average upper arm length (39.1 cm) [32], where obstacles can be detected with regular exploration.

### 3.4. HC-SR04 Results

In order to validate the accuracy of the ultrasonic interference detection system, a test where two ultrasonic devices emits pulses in a common area was made. In this case, interference between pulses can occur. The first ultrasound is a standard HC-SR04 without any modifications. This device emits pulses at random times. The second device is an eBAT prototype with phase modulation. The eBAT is tampered by the standard ultrasonic sensor, and the phase coding system should reduce the number of erroneous measurements.

The test included 692 measurements: some were made without interference, and some are tampered with. The expected distance is used to detect correct measurements and errors. Thus, if the measurement is close to the expected distance, it is assumed to be a correct measurement.

The results are shown in Table 1. In the first row, the eBAT is used as a standard ultrasonic device that just measures the echo time. In this case, with a number of measurements of 692, there are 637 correct measurements and 55 errors. In the second row, the modulation information included in the ultrasonic signal is used to discriminate the possible errors. In this case, the measurement of a correct distance can be classified as an error due to the lack of modulation. This can occur because the interference occurs at the same time. Using the information, every measurement can be classified in Correct Positives (CP), Correct Negatives (CN), False positives (FP), and False Negatives (FN). Correct Positives are the values correctly demodulated and with a correct distance. Correct Negatives are measurements incorrectly demodulated and with an incorrect distance. False Positives are measurements correctly demodulated but with an incorrect distance. False Negatives are measurements incorrectly demodulated but with a correct distance. As Table 1 shows, the number of errors is reduced from 55 to 7. There is only one false positive, so this assures the security of the user. The number of false negatives, where the distance is correct but the demodulation is not correct, is not representative. Thus, the ultrasonic information can be tampered with just at the same time that the echo is received, so it is a correct solution to invalidate that measurement.

In Table 2, the percentage error in the experiments is shown. The error is reduced from 7.95% to 1.01%, which shows a great improvement. It is more important to highlight that only one false positive is found, a 0.14% of the measurement. This test shows the improvement obtained in the device with a very low implementation costs. The results show better accuracy than any other ultrasonic transceiver and increased security and robustness.

This means it has a more than detection rate greater than 98% and a Bit Error Rate (BER) close to 0.01, which better than other studies [17,18] also using DPSK modulation and transmission over the air.

As a reference, for this experiment, the user can be warned about inaccurate data after 300 ms (three consecutive measurements). For an average blind walking speed of 0.6 m/s [33], only 18 cm are traversed by the subject.

## 4. eBAT User Interface

To solve the problems faced in the past, the ETA prototype was designed and developed after several iterations with feedback from blind persons and Orientation and Mobility (O&M) specialist trainers.

Our prototype includes two ultrasonic range sensors in a novel configuration. The first sensor is placed parallel to the horizon. The second is 45 degrees from the horizon, pointing to the ground. Thus, when the eBAT is placed on the chest, both obstacles at head level and arising from the floor can be detected. To keep the system affordable, an Arduino nano-microcontroller is used to drive the range sensors.

**Figure 15:**
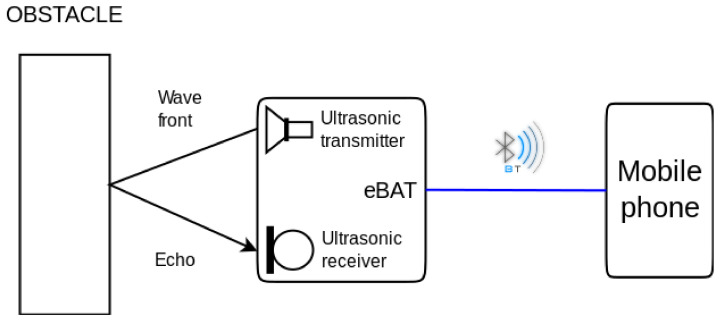
Block diagram of the eBAT working process. The range sensor emits an ultrasonic wave that is reflected by any obstacle in the path and received back as an echo. The eBAT then transmits the measured distance to a mobile phone connected through Bluetooth.

Information about the environment recorded by the device is transmitted to a mobile phone via Bluetooth connection, using a dedicated HC-06 module. A block diagram of the device is shown in Figure 15.

Therefore, the eBAT should be easy to use, as the only interaction needed by the users is through their own mobile phone. This is an advantage compared with other designs.

Estimated cost for the eBAT components is detailed in Table 3 and goes as low as 20 $.

At current stage of development, it is difficult to estimate the total cost of the eBAT. As first approximation, market-based pricing can be used, and a factor of two between the Cost of Goods Sold (COGS) and retail price can be applied [34,35]. Compared with similar devices (Section 2), a price of 100 $ is 3 times lower, and the COGS will be 100/2= USD 50. Deducting components cost, USD 30 is for other expenses like certification, taxes, and manufacturing.

From Section 3.1.1, the speed of sound at 20 °C (room temperature) is 343.65 m/s. To make calculations easier for the microcontroller, the number of microseconds it takes to do a 1 cm measurement (2 cm round trip) is used. This speed gives 58.2 micro seconds. As the microcontroller makes integer calculations faster, this value is rounded to 58.

One centimeter in 58 µs gives a speed of 344.8 m per second. That is the speed of sound for 22 °C, 0% humidity or 20 °C and 100% humidity, the final assumption for our device.

### 4.1. eBAT APP

For ETA devices, the electronics are an expensive and complex component that have to manage several measurements and provide complicated feedback to the user. Another factor is where to provide the haptic feedback.

Jones and Sarter [36] found that hairy skin is the most sensitive part to perceive the haptic feedback. The best location is the wrist and spine, followed by the arms. They also found that walking disturbs the perception in lower body sites, and if reaction time is critical, it is recommended that the haptic feedback is applied only in one site [37].

In order to minimize these factors, our design uses a mobile phone, with an application that takes care of the acquired data and configuration. Thus, costs can be contained and the user can choose the best location for the haptic feedback, providing it in one part of the body, as a low reaction time is preferred to avoid obstacles.

The developed application for Android OS receives the data about measured distances. Once in the phone, further calculations can be done using the high processing power of current devices. In addition, the configuration and the feedback to be provided can be easily carried out by the user. The approach of this study includes transformation of distance measured to the detected obstacles to a haptic feedback.

Haptic feedback is provided using vibrations pulses 100 ms in duration. Vibration frequency is then modulated using delays between pulses proportional to the spacing detected, including no delay and continuous vibration, and, for obstacles next to the sensor, a maximum of one pulse every second, which is the limit of the sensor. No vibration is provided when no detection is performed.

### 4.2. Path with Obstacles

**Figure 16:**
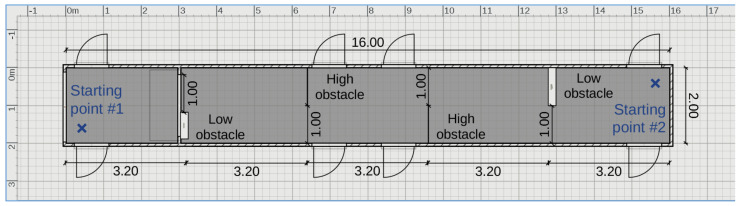
A diagram of the corridor with the obstacles in the path. The two possible orientations with starting point labeled with a blue cross and a “starting point” for each case. The total length was 16 m and with was 2 m. The space between obstacles was 3.2 m. On the left side, a double-leaf door is also drawn, which was left open during the tests.

To test the capabilities of the eBAT, an indoor path with obstacles was deployed along a corridor 16 m long by 2 m wide including four obstacles perpendicular to the direction of travel. Each obstacle was made from a foam core board of 1 m width by 1.2 m height. Two of them were attached to the ground and two at 0.8 m above the floor, hanging from the ceiling. A diagram of the corridor is shown in Figure 16.

Using both ends of the corridor gives two possible orientations for the path, with the same configuration of the obstacles (bottom-right; top-left; top-right; bottom left), as the distribution was symmetric.

After a short training period, (below 15 min as the device is simple and easy to operate), including explanations about eBAT prototype functioning, usage, how the feedback is provided, and a short description of the trip to be carried out, the path was traveled by the volunteers, the first time using the white cane as the only aid (without the eBAT) and then again with the eBAT and the white cane in the opposite way.

**Figure 17:**
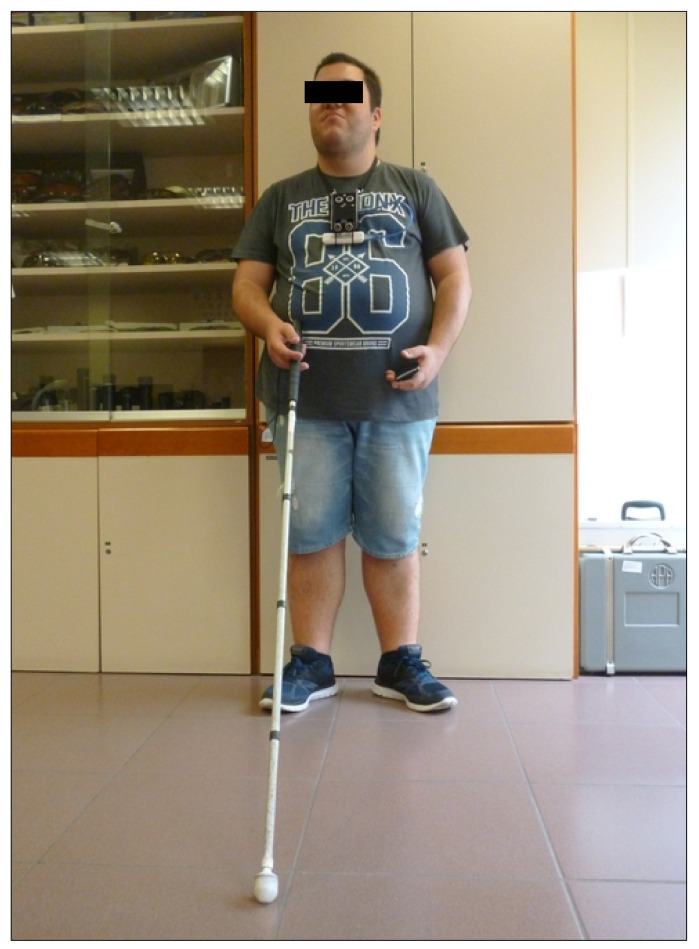
One of the volunteers wearing the eBAT (hanging from the neck) while receiving the short training to learn device operations.

The total number of involuntary contacts was measured in both cases. One of the subjects wearing the prototype is show in picture of the Figure 17.

### 4.3. Satisfaction Survey

Once the avoidance test was concluded, to check users satisfaction, a survey with five questions was performed. Questions included information about the size, usefulness, use of the mobile, safety and overall satisfaction. Answers were covered with a Likert scale from 0 (meaning “Nothing”) to 10 (“a lot”).

### 4.4. Results and Discussion

A total of 20 totally blind persons formed the experimental population, including 65% men and 35% women, with ages between 28 and 56 years old (Average 39.7; SD 8.42) and with three autonomy levels: autonomous familiar environment 5%, autonomous familiar environment + public transport if necessary 40%, autonomous in familiar and unfamiliar environment 55%. All of them use a white cane as their main mobility assistant.

**Figure 18:**
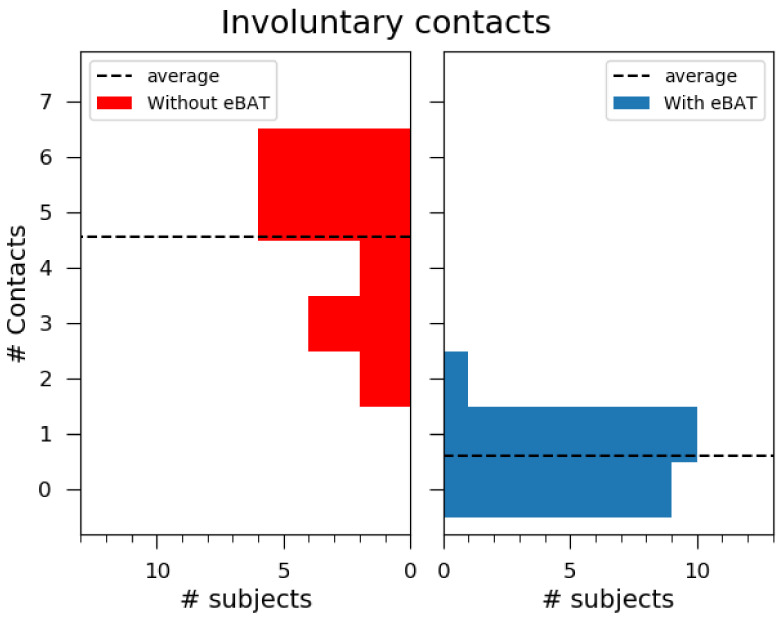
Histograms of number of involuntary contacts without (**left panel**) and with (**right panel**) the usage of the eBAT. Dashed lines indicate the average for each condition. It can be observed that the distribution without the eBAT is much lower, with the maximum at one involuntary contact.

The total number of involuntary contacts measured are shown in Figure 18.

The left panel (red color) shows a histogram of collisions that occurred without the use of the eBAT, and hte right panel (blue color) shows a histogram of collisions while wearing it. The dashed line shows the average for each side. It can be noted that the differences are quite notable between both sides.

From an average of 4.5 collisions without the use of the device, when the proposed ETA is included, it descends to below one. A T-test of the two samples [38] provides a value of 11.14 with a *p*-value $3.23\times 10^{-11}$ of each sample (with and without eBAT) with same average. It can be concluded that they are different populations.

Satisfaction results from the survey can be checked in Table 4. A lower average is associated with the size of the eBAT. In the other areas, it is greater than 7.5 over 10, including overall satisfaction of 8.3. It is also noticeable that the use of the mobile phone returns the best results in satisfaction. Regarding the dispersion of the answers, it is quite uniform among questions, with the exception, again, of the dimensions of the device, where it has the maximum value. The size of the prototype is already planned to be reduced in the following iterations.

To check the internal consistency of the survey answers, Cronbach’s alpha [39] was used, with a result of 0.71 (the range between 0.7 and 0.8 is usually stated as acceptable). Removing the size gives a value of 0.85, falling in the range of good.

Correlation test of the answers using Pearson’s r [40] obtained a value of 0.4 for autonomy and −0.5 with involuntary contacts compared with the overall satisfaction. The factor is not very large in any case but gives a result that volunteers with better autonomy and less involuntary contacts were more satisfied with the eBAT.

Other authors also performed satisfaction surveys with their prototypes. Katzschmann [14] obtained overall satisfaction of 82%, quite close to this study. Vorapatratorn [12] also stated a similar total score of 4.13 out of 5 (82.6%) but with very good results for the size of the iSonar: 4.67 out of 5 (93.4%). A prototype using a different ultrasonic range sensor [30] obtained that only 26.7% of the visually impaired scored its size as very satisfactory.

For already commercially available ETAs, Roentgen [33] also found good evaluations of the dimensions (4 out of 5). However, the effectiveness and safety (70%) are lower than the values expressed by the volunteers of this study (76.5 and 75.5% respectively), which is a prototype in an early development phase.

A summary comparing eBAT with other ETAS is shown in Table 5.

## 5. Conclusions

In this paper, the design of an aid system for blind people based on ultrasonic sensors is presented, focusing on high satisfaction of the users while keeping an assumption of a low budget.

There are several factors to be considered with an ultrasonic range sensor: accuracy, detection range, angle and price. Last one is key to maintaining a low cost. In addition, it should be tested whether or not the frequency is disturbing to guide dogs.

A temperature sensor could improve accuracy in the speed of sound estimation, but this value does not affect the measurements proportionally, so it can be skipped without appreciably reducing the mentioned improvements.

The design is made with standard electronic components and sensors, such as Arduino Nano, and low-cost HC-SR04 ultrasonic sensor. These devices are modified in hardware and software to improve its capabilities with a low-cost design.

Stability and measurement angle of the HC-SR04 have been tested. Noise is quite good over one thousand measurements spread over 15 min. Angle is enough to detect obstacles and not too high to detect other features not in the travel path. The measurement angle change with detection object size on average can fall within the value provided by the manufacturer.

A phase modulation in the ultrasonic pulses is included in order to detect interference between different ultrasonic emitters. This allows it to discriminate the received pulse information and select only the pulses emitted by the same circuit. This improves accuracy and security from tampering of other ultrasonic sources, reducing in our experiments the error rate from 7% to 1%. The False Positives is reduced to 0.1% of measurements. This modification is included in the ultrasonic system with no over cost, just with low-cost modifications of a standard ultrasonic device.

The eBAT prototype proves that with a budget as low as 20$, it is possible to support the totally blind reducing, involuntary contacts and improving safety in a controlled environment. Further research must be done in real pathways, both indoors and outdoors.

User satisfaction with the prototype is quite high, excluding the size of the device, which has to be improved in a future development. The usage of the mobile phone is stated as one of the strengths of the design, having the highest score in the satisfaction survey.

The requirements of the ETA have been accomplished as an effective low-budget aid using standard low-cost components that can be used as a complementary tool of the white cane.

## Figures and Tables

**Table 1:** Experiment results with two interference devices.

Experiment	Total	Correct Positives	False Positives	Correct Negatives	False Negatives
Measurement using only first pulse registered	692	637	55		
Measurement using phase coding to detect interference	692	631	1	54	6

**Table 2:** Error percentage in the experiment.

Experiment	Error Total (Errors)/N	False Positives/N
Measurement using only first pulse registered	7.95%	
Measurement using phase encodding to detect interferences	1.01%	0.14%

**Table 3:** Estimated cost of the eBAT based on components’ price.

Component	Units	Unit Price (USD)		Total Price ($)	
HC-SR04 ultrasonic range sensor	2	1		2	
Arduino nano board	1	2	0.5	2	0.5
HC-06 Bluetooth module	1	3		3	
Battery	1	6		6	
Case	1	5		5	
Other (cables, screws, etc.)	1	1	0.5	1	0.5
		Total		20	$

**Table 4:** Average and standard deviations for answers to the questions in the satisfaction survey.

Item (Answer from 0 to 10)	Average (Standard Deviation)
How adequate is the size of the eBAT?	4.10 (1.22)
How useful is the eBAT for mobility?	7.65 (0.91)
How much beneficial do you think the use of the mobile phone with the eBAT entails?	8.75 (0.70)
What level of safety do you think the use of eBAT brings?	7.55 (0.97)
What level of overall satisfaction have you had with the eBAT?	8.30 (0.95)

**Table 5:** Comparison of the eBAT with other ETAs.

Device	Price ($)	Sensors	Feedback	User Satisfaction (%)	Singular Capabilities
eBAT	100	2 × US	Haptic	83	Mobile phone; Self device detection
Miniguide	499	1 × US	Haptic/sound	80	Torch form factor
Ultracane	830	2 × US	Haptic	80	Enhanced white cane
Buzzclip	249	1 × US	Haptic	Not available	Simple to configure and use
iSonar	-	1 × US	Haptic/sound	83	Small and compact design
Kaltzman et al.	>1000	7 × IR	Haptic	82	Infrared; Feedback for each sensor

## Data Availability

Not applicable.

## Abbreviation

The following abbreviations are used in this manuscript:

eBAT	electronic Buzzer for Autonomous Travel
ETA	Electronic Travel Aids
RF	Radio-Frequency
ToF	Time of Flight
OTP	One Time Programming
PSK	Phase-Shift Keying
O&M	Orientation and Mobility
COGS	Cost of Goods Sold
OS	Operative System
SD	Standard Deviation

## References

[1] World Healh Organization (2010). Global Data on Visual Impairments.

[2] Dandona L., Dandona R. (2006). Revision of visual impairment definitions in the International Statistical Classification of Diseases. BMC Med..

[3] Lamoureux E.L., Hassell J.B., Keeffe J.E. (2004). The determinants of participation in activities of daily living in people with impaired vision. Am. J. Ophthalmol..

[4] Hill E.W., Ponder P. (1976). Orientation and Mobility Techniques: A Guide for the Practitioner.

[5] Dunai L., Fajarnes G.P., Praderas V.S., Garcia B.D., Lengua I.L. Real-time assistance prototype—A new navigation aid for blind people. Proceedings of the IECON 2010—36th Annual Conference on IEEE Industrial Electronics Society.

[6] Blasch B.B., Long R.G., Griffinshirley N. (1989). Results of a national survey of electronic travel aid use. J. Vis. Impair. Blind..

[7] Dunai L.D., Lengua I.L., Tortajada I., Simon F.B. Obstacle detectors for visually impaired people. Proceedings of the 2014 International Conference on Optimization of Electrical and Electronic Equipment (OPTIM).

[8] Elmannai W., Elleithy K. (2017). Sensor-Based Assistive Devices for Visually-Impaired People: Current Status, Challenges, and Future Directions. Sensors.

[9] Dakopoulos D., Bourbakis N. (2010). Wearable Obstacle Avoidance Electronic Travel Aids for Blind: A Survey. IEEE Trans. Syst. Man Cybern. Part C.

[10] Abreu D., Codina B., Toledo J., Suárez A. (2020). Validation of an eBAT as a mobility aid for blind people. Assist. Technol..

[11] Phillips G. (1998). The Miniguide Ultrasonic Mobility aid. In GDP Research, South Australia. http://www.gdp-research.com.au/minig_1.htm.

[12] Vorapatratorn S., Nambunmee K. (2014). iSonar: An obstacle warning device for the totally blind. J. Assist. Rehabil. Ther. Technol..

[13] Gayathri G., Vishnupriya M., Nandhini R., Banupriya M.M. (2014). Smart walking stick for visually impaired. Int. J. Eng. Comput. Sci..

[14] Katzschmann R.K., Araki B., Rus D. (2018). Safe Local Navigation for Visually Impaired Users With a Time-of-Flight and Haptic Feedback Device. IEEE Trans. Neural Syst. Rehabil. Eng..

[15] Ramadhan A. (2018). Wearable Smart System for Visually Impaired People. Sensors.

[16] Fok D., Polgar J.M., Shaw L., Jutai J.W. (2011). Low vision assistive technology device usage and importance in daily occupations. Work J. Prev. Assess. Rehabil..

[17] Li M., Kim Y.T. (2018). Feasibility Analysis on the Use of Ultrasonic Communications for Body Sensor Networks. Sensors.

[18] Yi D., Jin H., Kim M.C., Kim S.C. (2020). An Ultrasonic Object Detection Applying the ID Based on Spread Spectrum Technique for a Vehicle. Sensors.

[19] Glennie C.L., Carter W.E., Shrestha R.L., Dietrich W.E. (2013). Geodetic imaging with airborne LiDAR: The Earth’s surface revealed. Rep. Prog. Phys..

[20] Freaks E. (2016). Ultrasonic Ranging Module HC-SR04. https://cdn.sparkfun.com/datasheets/Sensors/Proximity/HCSR04.pdf.

[21] Ashihara K. (2007). Hearing thresholds for pure tones above 16 kHz. J. Acoust. Soc. Am..

[22] Heffner H.E. (1983). Hearing in large and small dogs: Absolute thresholds and size of the tympanic membrane. Behav. Neurosci..

[23] Marioli D., Narduzzi C., Offelli C., Petri D., Sardini E., Taroni A. (1992). Digital time-of-flight measurement for ultrasonic sensors. IEEE Trans. Instrum. Meas..

[24] Tsai W.Y., Chen H.C., Liao T.L. (2006). High accuracy ultrasonic air temperature measurement using multi-frequency continuous wave. Sens. Actuators A.

[25] Zuckerwar A.J. (2002). Handbook of the Speed of Sound in Real Gases.

[26] Hartigan J.A., Hartigan P.M. (1985). The Dip Test of Unimodality. Ann. Statist..

[27] Prattico F., Cera C., Petroni F. (2013). A new hybrid infrared-ultrasonic electronic travel aids for blind people. Sens. Actuators A.

[28] Mcgill R., Tukey J.W., Larsen W.A. (1978). Variations of Box Plots. Am. Stat..

[29] Mocanu B., Tapu R., Zaharia T. (2016). When Ultrasonic Sensors and Computer Vision Join Forces for Efficient Obstacle Detection and Recognition. Sensors.

[30] Lee C.L., Chen C.Y., Sung P.C., Lu S.Y. (2014). Assessment of a simple obstacle detection device for the visually impaired. Appl. Ergon..

[31] Kim K., Choi H. (2021). A New Approach to Power Efficiency Improvement of Ultrasonic Transmitters via a Dynamic Bias Technique. Sensors.

[32] Fryar C.D., Gu Q., Ogden C.L. Anthropometric Reference Data for Children and Adults; United States, 2007–2010. https://stacks.cdc.gov/view/cdc/12223.

[33] Roentgen U.R., Gelderblom G.J., de Witte L.P. (2012). User Evaluation of Two Electronic Mobility Aids for Persons Who Are Visually Impaired: A Quasi-Experimental Study Using a Standardized Mobility Course. Assist. Technol..

[34] Morris M.H., Morris G. (1990). Market-Oriented Pricing: Strategies for Management.

[35] DiResta R., Forrest B., Vinyard R. (2015). The Hardware Startup: Building Your Product, Business, and Brand.

[36] Jones L.A., Sarter N.B. (2008). Tactile Displays: Guidance for Their Design and Application. Hum. Factors.

[37] Karuei I., MacLean K.E., Foley-Fisher Z., MacKenzie R., Koch S., El-Zohairy M. (2011). Detecting Vibrations Across the Body in Mobile Contexts. Proceedings of the SIGCHI Conference on Human Factors in Computing Systems, Vancouver, BC, Canada, 7–12 May 2011.

[38] Welch B.L. (1947). The generalization of ‘Student’s’ problem when several different population variances are involved. Biometrika.

[39] Bland J.M., Altman D.G. (1997). Statistics notes: Cronbach’s alpha. BMJ.

[40] Pearson K. (1895). Note on Regression and Inheritance in the Case of Two Parents. Proc. R. Soc. Lond..

